# Obstetric outcomes after fresh *versus* frozen-thawed embryo transfers: A systematic review and meta-analysis

**DOI:** 10.5935/1518-0557.20180049

**Published:** 2018

**Authors:** Matheus Roque, Marcello Valle, Marcos Sampaio, Selmo Geber

**Affiliations:** 1 ORIGEN - Center for Reproductive Medicine, Rio de Janeiro, Brazil; 2 Universidade Federal de Minas Gerais, Belo Horizonte, Brazil; 3 ORIGEN - Center for Reproductive Medicine, Belo Horizonte, Brazil

**Keywords:** Obstetric outcome, fresh embryo transfer, frozen-thawed embryo transfer, placenta

## Abstract

**Objective:**

To evaluate if there are differences in the risks of obstetric outcomes in IVF/ICSI singleton pregnancies when compared fresh to frozen-thawed embryo transfers (FET).

**Methods:**

This was a systematic review and meta-analysis evaluating the obstetric outcomes in singleton pregnancies after FET and fresh embryo transfer. The outcomes included in this study were pregnancy-induced hypertension (PIH), pre-eclampsia, placenta previa, and placenta accreta.

**Results:**

The search yielded 654 papers, 6 of which met the inclusion criteria and reported on obstetric outcomes. When comparing pregnancies that arose from FET or fresh embryo transfer, there was an increase in the risk of obstetric complications in pregnancies resulting from FET when compared to those emerging from fresh embryo transfers in PIH (aOR 1.82; 95% CI 1.24-2.68), pre-eclampsia (aOR 1.32, 95% CI 1.07, 1.63), and placenta accreta (aOR 3.51, 95% CI 2.04-6.05). There were no significant differences in the risk between the FET and fresh embryo transfer groups when evaluating placenta previa (aOR 0.70; 95% CI 0.46-1.08).

**Conclusion:**

The obstetric outcomes observed in pregnancies arising from ART may differ among fresh and FET cycles. Thus, when evaluating to perform a fresh embryo transfer or a freeze-all cycle, these differences found in obstetric outcomes between fresh and FET should be taken into account. The adverse obstetric outcomes after FET found in this study emphasize that the freeze-all policy should not be offered to all the patients, but should be offered to those with a clear indication of the benefit of this strategy.

## INTRODUCTION

Today, nearly one in six couples faces fertility issues, as they fail to achieve a clinical pregnancy even after regular copulation (Boivin *et al*., 2007; Zegers-Hochschild *et al*., 2009). Consequently, couples are turning to assisted reproductive technology (ART) to become pregnant, which will hopefully result in the birth of a healthy baby. In 1983, the first frozen-thawed embryo was transferred by Trounson, which resulted in a successful pregnancy (Trounson & Mohr, 1983). Since then, continuous advancements in cryopreservation techniques have been made and at present, the quality and potential for frozen-thawed embryo implantation is comparable to those of fresh embryos (Herrero *et al*., 2011; Shapiro *et al*., 2010).

Although fresh embryo transfer is still the norm in most *in vitro* fertilization (IVF) treatments, as it involves a shorter process that leads to pregnancy, this method is related to increased hormone levels due to controlled ovarian stimulation (COS). The supra-physiologic hormonal levels observed during COS results in a suboptimal uterine environment that may negatively impact embryo implantation and placentation, eventually culminating to untoward obstetrical and perinatal outcomes (Imudia *et al*., 2012; Kalra & Molinaro, 2008; Kalra *et al.*, 2011; Mainigi *et al*., 2016; Roque *et al*., 2017). Conversely, FET cultivates better environmental conditions within the uterus during embryo transfer, leading to improved endometrial receptivity (Barnhart, 2014; Weinerman & Mainigi, 2014). This better uterine environment may be related with better placentation during a FET cycle, leading to improved obstetric outcomes when compared to fresh transfer cycles (Maheshwari *et al*., 2012; Roque *et al*., 2015b; Shapiro *et al*., 2013). However, some studies have also shown that FET may have possible adverse effects on obstetric outcomes (Sazonova *et al*., 2012; Spijkers *et al*., 2017). Births from singleton ART pregnancies following FET have been associated with high birth weights, although there was a lower risk of preterm births when compared to fresh transfer cycles (Maheshwari *et al*., 2012; Spijkers *et al*., 2017; Wennerholm *et al*., 2013), a finding that highlights the impact of the clinical procedure itself, and not maternal characteristics, on these outcomes (Pinborg *et al*., 2014). Recently published meta-analysis comparing obstetric outcomes in pregnancies after fresh and FET did not report major obstetric outcomes such as pregnancy-induced hypertension (PIH), pre-eclampsia, placenta previa, and placenta accreta (Maheshwari *et al*., 2012; Pinborg *et al*., 2013).

To further examine the obstetric outcomes in singleton ART pregnancies, we performed a systematic review and meta-analysis of the available literature to compare the effects of FET and fresh embryo transfer on some major obstetric complications after IVF cycles that have not been reported in previous meta-analyses.

## MATERIALS AND METHODS

This systematic review and meta-analysis was carried out in compliance with the Preferred Reporting Items for Systematic Reviews and Meta-Analyses (PRISMA) guidelines. Approval from the institutional review board was not undertaken because all the data was gathered from previously published papers.

### Inclusion and exclusion criteria

We performed a systematic review and meta-analysis of observational studies. We carried out an extensive literature search in PubMed, EMBASE, and Cochrane databases from its inception through October 2015. We included only English-language papers and excluded conference abstracts if the full articles of the same study were not available. We also excluded studies that were performed without a control group. We used different search terms for obstetric outcomes in singleton pregnancies (e.g., "Obstetric outcomes", "Obstetric complications", "Pregnancy-induced hypertension", "Pre-eclampsia", "Placenta previa", and "Placenta accreta", "fresh embryo transfer", "frozen-thawed embryo trasnfer") and included articles comparing fresh embryo transfer with FET. We also reviewed the references of the selected studies and reviews to explore additional references. Only studies that provided the adjusted odds ratio (aOR) were included. The selection criteria are described in Table 1.

**Table 1:** PICOs - Population, Intervention, Comparison, and Outcomes of interest.

Target population	Singleton pregnancies of women undergoing ART
Intervention	Fresh embryo transfer vs Frozen embryo transfer
Outcome measure	•Pregnancy-induced hypertension •Pre-eclampsia •Placenta previa •Placenta accrete
Design	Cohorts or Case-control

### Eligibility criteria and data extraction

In a first screening, two independent authors (MR, MV) assessed all of the abstracts retrieved from the search, and then they obtained the full manuscripts of citations that fit the inclusion criteria. At first, the studies were screened based on the information available in the abstract and title. In the second phase, only those articles that were screened in the first phase were evaluated; at this point, they were assessed for their eligibility to be included in this study based on our aforementioned screening criteria. In the third phase, complete articles were assessed to define their eligibility for the meta-analysis. The authors considered study eligibility, assessed quality, and extracted data solving discrepancies by agreement, and if needed, reaching a consensus with a third author (SG). All authors critically analyzed the summarized results.

The original studies included here reported on the comparisons made between the outcomes of fresh embryo transfer and FET for singleton pregnancies following ART. Studies examining only frozen and donor oocytes were excluded.

### Outcome measures

The outcomes were the development of pregnancy induced-hypertension (PIH), pre-eclampsia, placenta previa, and placenta accreta.

### Risk of Bias assessment

To access the risk of bias of the studies included, we followed the ROBINS - I: the Risk Of Bias In Non-randomized studies of interventions (Sterne *et al*., 2016). The studies were evaluated on bias: due to confounding; in selection of participants; in classification of interventions; due to deviations from intended interventions; due to missing data; in measurements of outcomes; in selection of reported results. After that, an overall bias risk for each study was determined as low, moderate, serious, or critical.

### Data extraction and analysis

To determine the pooled effect of each variable, we used a Mantel-Haenszel model and applied the fixed-effects model. The adjusted odds ratio (aOR) accompanied by the 95% confidence intervals (CIs) were calculated. Statistical significance was set at a *p* value <.05. We evaluated the degree of variation across studies attributable to heterogeneity with the *I^2^* statistic. When the heterogeneity was greater than 50% (*I^2^* > 50%), we applied the random-effects model (Higgins *et al*., 2003). We conducted a meta-analysis using Review Manager 5 Software (Cochrane Collaboration).

## RESULTS

**Figure 1:**
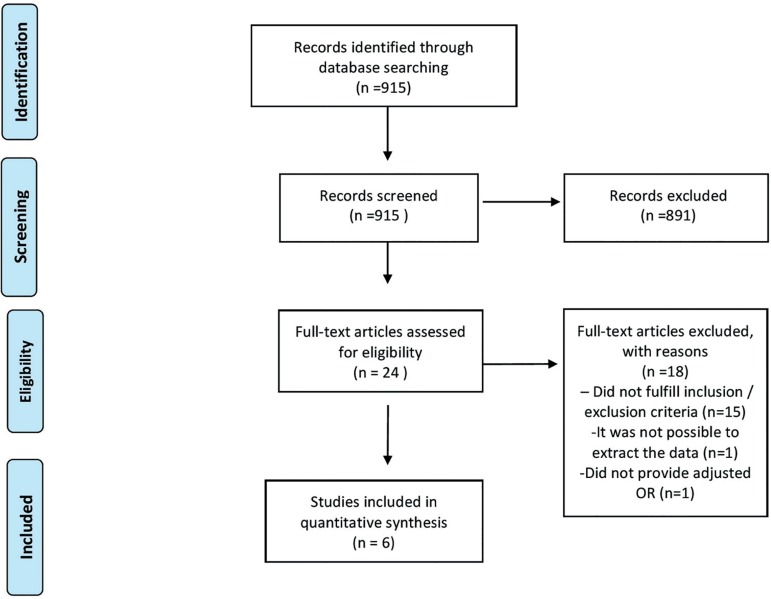
Flowchart for the trial identification and selection process.

Our electronic search retrieved 915 articles but 891 were excluded at the title/abstract screening. One or both reviewers considered the remaining 24 studies eligible. Among these, eighteen articles were excluded because they did not fulfill the inclusion criteria, as they did not report the included outcomes in this review (Aytoz *et al*., 1999; Kalra *&* Molinaro, 2008; Wennerholm *et al*., 2013; Belva *et al.*, 2008; Henningsen *et al*., 2011; Ishihara *et al*., 2014; Kato *et al*., 2012; Källén *et al*., 2005a, Li *et al.*, 2014; Pelkonen *et al*., 2010; Pinborg *et al*., 2010; Schwarze *et al.*, 2015; Shih *et al*., 2008; Wang *et al*., 2005; Wennerholm *et al*., 1997), did not provide the adjusted OR (Imudia *et al*., 2013), and the last excluded study because it did not present the comparison between fresh and FET cycles (Källén *et al*., 2005b). Six articles met inclusion criteria and were included in this review (Healy *et al*., 2010; Ishihara *et al*., 2014; Kaser *et al*., 2015; Opdahl *et al*., 2015; Rombauts *et al*., 2014; Sazonova *et al*., 2012) (Figure 1 - Flowchart). The characteristics of included articles are described in Table 2.

**Table 2:** Characteristics of the included studies.

Study	Study Design	Country	Period	Outcome included in the meta-analysis	FET vs Fresh aOR (95% CI)
Healy *et al*., 2010	Retrospective cohort (population-based registry - Victoria)	Australia	1992-2004	Placenta previa	0.71 (0.51, 1.00)
Ishihara *et al*., 2014	Retrospective cohort (nationwide registry)	Japan	2008-2010	Pregnancy induced hypertension Placenta previa Placenta accreta	1.58 (1.35, 1.86) 0.91 (0.70, 1.19) 3.16 (1.71, 6.23)
Kaser *et al*., 2015	Case-control study (single-center analysis)	United States	2005-2011	Placenta accreta	4.54 (1.65, 12.47)
Opdahl *et al*., 2015	Retrospective cohort (nationwide registry)	Denmark, Norway and Sweden	1988-2007 (Sweden and Norway) 1997-2007 (Denmark)	Pregnancy induced hypertension	2.39 (1.48, 3.86)
Rombauts *et al.,* 2014	Retrospective cohort (single-center analysis)	Australia	2006-2012	Placenta previa	1.13 (0.61, 2.10)
Sazonova *et al*., 2012	Retrospective cohort (nationwide registry)	Sweden	2002-2006	Pre-eclampsia Placenta previa	1.32 (1.07, 1.63) 0.32 (0.19, 0.54)

### Pregnancy-induced hypertension

**Figure 2:**
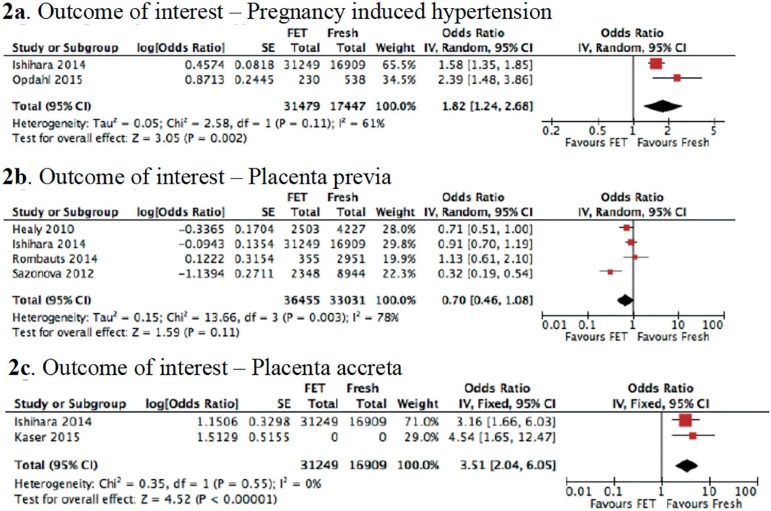
Summary of a meta-analysis (presenting the adjusted odds ratios [aOR] and 95% confidence intervals [CI]) examining the secondary obstetric outcomes in singleton ART pregnancies after FET and fresh embryo transfer. **a:** Summary of a meta-analysis of two studies (presenting the adjusted odds ratios [aOR] and 95% confidence intervals [CI]) examining PIH as an obstetric outcome in singleton ART pregnancies after FET and fresh embryo transfer. **b:** Summary of a meta-analysis of four studies (presenting the adjusted odds ratios [aOR] and 95% confidence interval [CI]) assessing placenta previa as an obstetric outcome in singleton ART pregnancies after FET and fresh embryo transfer. **c:** Summary of a meta-analysis of two studies (presenting the adjusted odds ratios [aOR] and 95% confidence intervals [CI]) evaluating placenta accreta as an obstetric outcome in singleton ART pregnancies after FET and fresh embryo transfer.

Two studies were included in this analysis (Ishihara *et al*., 2014; Opdahl *et al*., 2015). A total of 48,926 cases were eligible for inclusion in the analysis of PIH in singleton pregnancies after fresh embryo transfer and FET. Of these cases, 31,479 singleton pregnancies resulted from the transfer of frozen-thawed embryos, and 17,447 singleton pregnancies occurred after the transfer of fresh embryos. We found that the risk of developing PIH increased in the FET group compared to the fresh embryo transfer group (aOR: 1.82; 95% CI: 1.24-2.68; *I^2^* = 61%; *p*=0.002) (Figure 2a).

### Pre-eclampsia

There was only one study (Sazonova *et al*., 2012) evaluating this outcome. A total 2,348 singleton pregnancies after FET and 8,944 after fresh cycles were evaluated in the study. There was a higher risk of pre-eclampsia (aOR 1.32, 95% CI 1.07, 1.63) in singleton pregnancies after FET than after fresh cycles.

### Placenta previa

There were 4 studies included in this analysis (Healy *et al*., 2010; Ishihara *et al*., 2014; Rombauts *et al*., 2014; Sazonova *et al*., 2012). A total of 69,486 pregnancies from four studies were included. It was found that 36,455 singleton pregnancies were reported after the transfer of frozen-thawed embryos, and 33,031 emerged following the transfer of fresh embryos. There were no significant differences in the risk of placenta previa development between the fresh embryo transfer and FET groups (aOR: 0.70; 95% CI: 0.46-1.08; *I^2^* = 78%; *p* = 0.11) (Figure 2b).

### Placenta accreta

Two studies were included in this outcome (Ishihara *et al.,* 2014; Kaser *et al*., 2015). We found that the risk of placenta accreta development increased significantly in the FET group compared to the fresh embryo transfer group (aOR: 3.51; 95% CI: 2.04-6.05; *I^2^* = 0%; *p* < 0.00001) (Figure 2c).

## DISCUSSION

In this study, we performed a systemic review and meta-analysis of the effect of FET and fresh embryo transfer on the risks of developing major obstetric complications in singleton pregnancies following the use of ART. To our knowledge, the current study is the first systematic review and meta-analysis comparing the adjusted data of PIH, pre-eclampsia, placenta previa and placenta accreta in singleton pregnancies after fresh and FET cycles.

Elucidating the IVF effects on obstetric outcomes in singleton pregnancies is of utmost importance in the field of reproductive medicine and mother-child health. Successful IVF depends not only on the quality of the embryo (Lee *et al*., 2017), but also on endometrial receptivity and the environmental conditions of the uterus during the pre-implantation period (Barnhart, 2014; Schoolcraft *et al*., 2017; Shapiro *et al*., 2014). With continuing research in this area, updated knowledge on endometrial-embryo interactions can help researchers and clinicians better understand the positive and negative outcomes of IVF. When selecting an ART method that employs fresh embryo transfer, the primary concern is the use of COS, which can damage the endometrial and uterine environment (Roque, 2015a). FET cycles are performed in a physiological uterine environment and this may be the reason that some studies observed better IVF outcomes following FET than after fresh embryo transfer (Shapiro *et al*., 2014, 2011a,b; Chen *et al*., 2016; Roque *et al*., 2015b).

The application of FET has continuously increased over the last few years (Pereira & Rosenwaks, 2016) by as much as 82.5% in 2006-2012 nationally in USA, while globally, its application has increased by 27.6% from 2008-2010 (Dyer *et al*., 2016). Recent studies highlighted that FET is associated with better safety and obstetric outcomes when compared to fresh transfer cycles (Shapiro *et al*., 2013; Maheshwari *et al.*, 2013; 2016). However, some studies have also intimated about the fact that FET may have possible adverse effects on obstetric outcomes (Sazonova *et al*., 2012; Spijkers *et al*., 2017). In the present study, we found an increase in the risk of PIH, pre-eclampsia and also placenta accreta in singleton pregnancies after FET when comparing to fresh embryo transfers. There were no differences in the risk of placenta previa when comparing fresh to FET.

Pregnancies following FET had significantly higher odds for developing obstetric outcomes such as pregnancy-induced hypertension (PIH) and placenta accreta (Ishihara *et al*., 2014). Recent studies have revealed that the PIH risk is increased in singleton ART pregnancies when compared with spontaneously conceived singleton pregnancies (Jackson *et al*., 2004; Thomopoulos *et al*., 2013). Importantly, the higher risk of PIH in pregnancies that result from FET may not be entirely associated with maternal characteristics. A study on the risk of hypertensive disorders suggested that the risk of PIH development is higher in pregnancies following FET compared to fresh embryo transfer, even when the same mother is considered (Opdahl *et al*., 2015). The authors also wondered whether there were cases where women had more than one embryo transferred, as this might also contribute to the lowered risk of PIH. Similarly, a Japanese study conducted in 2008-2010 indicated that the risk of PIH was higher in pregnancies after FET than after fresh embryo transfer (Ishihara *et al*., 2014). During the present meta-analysis, we observed similar outcomes; we found that the risk of PIH in singleton ART pregnancies increased after FET when compared with fresh embryo transfer (aOR = 1.82; 95% CI 1.24-2.68; *p* = 0.002).

Placenta accreta is a very rare complication in pregnancies that result from ART. This type of placental development can lead to serious maternal outcomes and subsequent hysterectomy. One study reported that there were higher odds of developing placenta accreta following FET (Ishihara *et al*., 2014). Further, a multivariate analysis exploring FET as a risk factor for placenta accreta was carried out, and the authors found that FET is a strong independent risk factor for placenta accreta, even after controlling for those conditions that are known risk factors for this condition and other possible complications unique to ART (Kaser *et al*., 2015). The authors further confirmed that the increased risk of placenta accreta is directly associated with factors related to FET and not with patient characteristics. They proposed that the possible mechanisms underlying the increased risk of this pregnancy complication might include lower serum E2 levels and a thinner endometrial lining in FET cycles, which both contribute to uncontrolled growth of the extravillous trophoblast into the myometrium (Kaser *et al*., 2015). Similar to the previous studies, we also found that there are increased outcomes of placenta accreta in singleton pregnancies after FET than after fresh embryo transfer (aOR 3.51; 95% CI 2.04-6.05; *p*<0.001). It is noteworthy that higher serum E2 levels are associated with the risk of fetal growth restriction and pre-eclampsia. Hence, it is necessary to manipulate the level of serum E2 for ART cycles, and further studies are required in this direction.

Various reports have suggested that there is a higher rate of placenta previa in ART singleton pregnancies when compared with spontaneous pregnancies (Healy *et al*., 2010; Källén *et al*., 2005a,b; Jackson *et al*., 2004; Romundstad *et al*., 2006; Schieve *et al.*, 2007). Few studies have also performed comparisons of the risk of placenta previa in cryopreservation and fresh cycles (Wikland *et al*., 2010; Healy *et al*., 2010; Pelkonen *et al*., 2010). A lower rate of placenta previa was found in singleton pregnancies following cryopreservation cycles than in fresh cycles (Sazonova *et al*., 2012). Conversely, some studies indicated that there were no associations between the risk of placenta previa and the type of embryo transfer method used (Healy *et al*., 2010; Ishihara *et al*., 2014; Rombauts *et al*., 2014). In our meta-analysis, we found that there was no significant variations in the risk of placenta previa in singleton pregnancies after FET and fresh cycles (aOR = 0.70; 95% CI 0.46-1.08; *p* = 0.11).

Detailed studies are needed to better understand the effects of COS and cryopreservation on the health of mothers and their offspring. Our study is based on observational studies, making it subject to biases. Moreover, in this study it is not possible to evaluate between the different types of cryopreservation protocols (slow freezing or vitrification) and also the embryo developmental stage (cleavage or blastocyst). The findings of our study should be considered with caution as the overall quality of evidence is low to moderate. Although this study included few papers, it raises concerns about the risk of some major obstetric complications after FET. These findings are important to be taken into account when evaluating to perform a fresh embryo transfer or freeze-all cycle.

In conclusion, the obstetric outcomes observed in pregnancies arising from ART may differ among fresh and FET cycles. Thus, when evaluating to perform a fresh embryo transfer or a freeze-all cycle, these differences observed in obstetric outcomes between fresh and FET should be taken into account. The adverse obstetric outcomes after FET observed in this study emphasize that the freeze-all policy should not be offered to all the patients, but should be offered to those with a clear indication of the benefits of such strategy.
